# Effect of medically assisted reproduction (MAR) and pregnancy planning on Bayley-III Screening Test subscales in preterm infants at 12 months of corrected age: a cross-sectional study

**DOI:** 10.1186/s13052-022-01259-w

**Published:** 2022-05-08

**Authors:** Anna Szabina Szele, József Mihály Gáll, Beáta Erika Nagy

**Affiliations:** 1 Faculty of Medicine, Pediatric Psychology and Psychosomatic Unit of the Department of Pediatrics, Kálmán Laki Doctoral School, University of Debrecen, 98. Nagyerdei Boulevard, 4032 Debrecen, Hungary; 2 Faculty of Informatics, Department of Applied Mathematics and Probability Theory, University of Debrecen, Debrecen, Hungary

**Keywords:** Preterm infants, Low birth weight, Pregnancy planning, Medically assisted reproduction, Neurodevelopment

## Abstract

**Background:**

The association between medically assisted reproduction and pregnancy planning on overall developmental outcomes of infants has been studied in relatively few studies. The availability of accurate tools for the early detection of developmental delays is a major issue. The purpose of this study is describing the average neurodevelopment of preterm infants and assessing the association between medically assisted reproduction, pregnancy planning and neurodevelopmental outcomes among Hungarian preterm infants.

**Methods:**

Cross-sectional study of preterm infants with gestational age < 37 weeks (*N* = 171) who underwent developmental assessment using Bayley-III Screening Test (Bayley Scales of Infant and Toddler Development Screening Test – Third Edition) in five developmental domains (cognitive, receptive and expressive language, fine and gross motor) at 12 months of corrected age. We examined the developmental pattern of infants and the potential associations between medically assisted reproduction, pregnancy planning and Bayley-III Screening Test subscales. Information on the mode of conception and pregnancy planning was obtained through parental anamnesis.

**Results:**

Concerning the risk for developmental delay, the language and motor subscales were the most affected. Examination of the mode of conception and developmental outcomes revealed higher neurodevelopmental skills in infants who were conceived through medically assisted reproduction. Significantly lower cognitive, receptive and expressive language skills were found to be associated with unplanned pregnancies. Multi-way analysis of variance was conducted in order to assess the impact of the mode of conception and pregnancy planning on subscales.

**Conclusions:**

We cannot unambiguously conclude that MAR and pregnancy planning had a solely positive effect on the development of preterm infants at 12 months of corrected age, but our results are vital for the design and implementation of further research.

## Background

In spite of the development of medical sciences, the number of preterm births is increasing worldwide. Every year approximately 15 million babies are born preterm (before 37 weeks of pregnancy); often associated with an overlapping perinatal risk, low birth weight (< 2500 g). Preterm birth is not only the leading cause of death among children under the age of 5 [1], but it is responsible for a number of neonatal morbidities that can cause long-term difficulties such as cerebral palsy, vision and hearing impairment, social and emotional problems [2–4].

The presence of neonatal morbidities like bronchopulmonary dysplasia (BPD), necrotizing enterocolitis (NEC), intraventricular haemorrhage (IVH), retinopathy of prematurity (ROP), chronic lung disease (CLD), and sepsis may associate with lower neurodevelopmental outcomes [5–7]. BPD, NEC, IVH, and ROP are particularly linked to the prematurity. BPD is a chronic lung disease of immature new-borns that is commonly preceded by severe condition of respiratory distress syndrome. According to the currently definition, a diagnosis of BPD can be made if the preterm infant requires oxygen therapy for a minimum of 28 days, which is still required at the 36th gestational week of the corrected age. NEC is a severe gastrointestinal disease, inflammation in the intestinal tract, which can lead to necrosis and perforation. IVH or germinal matrix haemorrhage is one of the leading causes of neonatal mortality and nervous system damage, classified along grades I-IV, which are differentiated by the extent of bleeding. While the ROP is an eye disorder, the vasoproliferative disease of the retina, which is classified in five stages (I-V) from mild to severe visual impairment [8, 9]. Congenital heart defects (CHD), as atrial septal defect (ASD), ventricular septal defect (VSD) and patent ductus arteriosus (PDA) which can aggravate the clinical conditions of preterm new-borns should also be mentioned. ASD means a defect in the septum that separates the two upper chambers of the heart, while VSD is a hole in the septum that separates lower chambers. PDA is resulted from the failure of normal closure of ductus arteriosus, which is a vessel between the aorta and the pulmonary artery [10, 11]. According to literature findings, preterm infants have significantly lower cognitive, language and motor skills in the early years of life [12, 13]. These morbidities can influence both the early and later life of preterm children; determine the quality of life of them e.g. BPD can cause poor neurodevelopmental outcomes, long-term pulmonary diseases, like airway hiperreactivity and obstruction, which persist into adolescence and adulthood [14–16]. NEC commonly cause poor growth and neurodevelopmental impairment [16, 17] and is associated with worse developmental characteristics than prematurity alone [18]. The severity of ROP and IVH may associates with lower intelligence even in the early years of life [19]; higher grades of IVH are also associated with worse motor functioning, and poorer academic skills at school-age children [20].

Higher preterm birth rates and lower birth weight can often be identified in unplanned and children conceived through assisted reproduction [21, 22]. Next to the growing of the levels of involuntary childlessness, the number of babies born after assisted reproductive technologies is currently rising. Since 1978, the birth of the first ART baby, more than 5 million children have been born via ART worldwide [23, 24]. According to the latest ICMART (International Committee Monitoring Assisted Reproductive Technologies) world report, concerning ART treatments, Europe is the most affected, followed by Asia and North America [25]. The European Atlas of Fertility Treatment Policies provide us an extensive look into the fertility policies of 43 European Countries. According to its results, most countries have dedicated laws on reproductive technologies or a national register. In five countries, ART and intrauterine insemination (IUI) are allowed for both infertile heterosexual couples, single women and same sex couples. 41 countries provide insemination with donor sperm, egg donation is allowed in 38, the simultaneous donation of sperm and egg is permitted in 32, and embryo donation is allowed in 29 countries. Funding was also found to be diverse, only 12 countries provide up to six funded IUI interventions. Three countries (Greece, Serbia and Slovenia) offer up to six funded IVF/ICSI interventions, while 35 countries offer partially funding (e.g. Hungary). Regarding the fertility policies in Europe, Belgium, The Netherlands, France, Portugal, Finland, Norway, Croatia, Hungary and the United Kingdom are in the forefront [26, 27].

To resolve the problem of infertility and involuntary childlessness, assisted reproductive technologies offer a set of techniques (among others e.g. intracytoplasmic sperm injection (ICSI), in vitro fertilization (IVF), frozen embryo transfers) [23, 24]. However, there are some common procedures, which cannot be classified into the ART treatments, such as ovulation induction or artificial insemination. This fact necessitated the development of a broader concept, medically assisted reproduction (MAR) [23]. MAR techniques include four main procedures: ovulation induction, artificial insemination, IVF and ICSI [21]. The number of children born after MAR is also high, more than five million [21, 28]. These babies are more exposed to health risks and adverse perinatal outcomes than their naturally conceived peers. Transferring more than one embryo can lead to a higher rate of multiple pregnancies, increased risk of preterm birth and low birth weight [21]. According to the meta-analysis of Djuwantono et al. [29], children born after assisted reproductive technology show higher risk of cerebral palsy, while the risk of autism spectrum disorder and intellectual disability is higher in ICSI children. In many cases, the subfertility and advanced maternal age alone can mean a higher risk for worse outcomes [30, 31]. Monitoring and assessment of the developmental differences of these children from naturally conceived peers are essential.

Pregnancy planning is generally related to prenatal and postnatal behaviour of parents [32]. According to the global estimation of Bearak et al. [33], the rate of unplanned/unintended pregnancies was 44% in 2010–2014. These pregnancies can be the consequence of socioeconomic inequality and lead to lower employment resources and education [33, 34]. In case of unplanned, unwanted pregnancies, we often find poorer perinatal outcomes, higher rates of preterm birth, lower birth weight [22], lower paternal support [35] and difficulties in breastfeeding [36]. These factors can directly and indirectly influence the child's development. Kenyhercz et al. [37] highlighted the importance of breastfeeding and positive maternal emotional state before and after birth in the quality of life of 2-year-old preterm children. However, epidemiological research into the association between pregnancy planning and later development of a child is scarce.

The objective of this study:

1. Description of the neurodevelopmental outcomes (cognitive, receptive and expressive language, fine and gross motor skills) of preterm infants at 12 months of corrected age in a Hungarian sample.
2. Examination of the potential association between medically assisted reproduction, pregnancy planning and neurodevelopmental outcomes of preterm infants at 12 months of corrected age in a Hungarian sample.

## Methods

### Sample

In this work 171 preterm (< 37 weeks) infants at 12 months of corrected age were examined at the *Pediatric Psychology and Psychosomatic Unit of the Department of Pediatrics of the University of Debrecen* between December 2017 and May 2019. In our sample the main inclusion criteria was the prematurity and the undergoing of all the five subscales of Bayley-III Screening Test. Note that, everyone in the sample happened to have LBW, though it was not an inclusion criteria. Every subject who was born prematurely at the *Department of Obstetrics and Gynecology of the University of Debrecen* between December 2016 and May 2018 (*N* = 640) was invited to take part in the examination, except for infants with severe damage and those how have died. A total of 469 subjects were excluded (neonatal death, severe damage, rejection of participation) from the examinations. 169 infants from our sample were delivered in the same maternity ward, at the *Department of Obstetrics and Gynecology of the University of Debrecen*, while there were only two who were outborn (born at home and in another hospital). Our sample covers all available cases in the Eastern region of Hungary. Correction for prematurity was made based on the instructions of the Screening Test Manual [38]. The study was approved by the Medical Research Council of Hungary in compliance with the Ethical Principles of the World Medical Association Declaration of Helsinki. From subjects’ parents we asked a written informed consent.

### Bayley-III Screening Test (Bayley Scales of Infant and Toddler Development Screening Test – Third Edition)

Developmental psychodiagnostic tools play an important role in the identification of developmental delays. BSID-III (Bayley Scales of Infant and Toddler Development – Third Edition) – the advanced version of BSID-II (Bayley Scales of Infant Development – Second Edition) – is currently the most advanced and widespread developmental tool for infants and toddlers [39]. Due to its two indices (mental and motor), BSID-II was not able to distinguish developmental delay in the cognitive and language, or the fine and gross motor skills [40]. Development of the Third Edition of Bayley-III was a great leap forward. It measures the early developmental performance of infants and toddlers between 1 and 42 months in five distinct subscales (cognitive, receptive and expressive language, fine and gross motor) [38, 39]. In Hungary, both the diagnostic and screening tests were adapted and standardized in 2017. In our study, we used the Bayley-III Screening Test to determine if the child is developing appropriately or more comprehensive assessment is needed. By administering the Screening Test, high, moderate and low risk for developmental delays can be distinguished. To classify the performance, the child’s total score is compared to given norms. For infants aged 12 months and younger, it takes approximately 15 to 25 min to complete [38, 41].

### Developmental characteristics; examination of the mode of conception and pregnancy planning

Developmental characteristics (gestational age, birth weight), were obtained from the clinical database of the *Department of Pediatrics of the University of Debrecen*. Information about the mode of conception (conceived by spontaneous or MAR (ovulation induction, artificial insemination, IVF and ICSI)) and pregnancy planning (planned – unplanned) was obtained based on a maternal anamnesis.

### Statistical analysis

Several descriptive statistics were calculated for infants’ main characteristics (perinatal and socio-demographic data) and developmental performance. Independent-samples (Student’s) t-test was used to compare the means of neurodevelopment of infants conceived by MAR or naturally and planned or unplanned infants. If the standard deviations of the compared populations did not coincide, Welch’s t-test was used, instead, as the natural alternative test. In addition, a non-parametric alternative, the Mann–Whitney test, was also carried out to analyse the difference in the distributions of the compared groups for the key variables (Bayley-III Subscales). Note that, the latter one is fairly useful in case of smaller sample size. Comparison of the distribution of some nominal variables (e.g. perinatal and socio-demographic ones) again between MAR and naturally conceived as well as planned and unplanned groups were done by χ^2 test. To examine whether mode of conception and pregnancy planning were the most influencing factors for positive or negative changes in cognitive, language (receptive and expressive) and motor (fine and gross) development, analysis of variance (ANOVA) was performed with several factors (‘multi-way’) including possible interactions as well. We tested whether the independent variables in neurodevelopmental outcomes remain significant or not, in addition to other explanatory factors. For all statistical analysis, *IBM SPSS Statistics v25* was used.

## Results

### Descriptive statistics (mean and standard deviation)

In what follows, notations M, SD and n are used for sample mean, sample standard deviation and sample size, respectively. All infants participating in the study (*n* = 171) were Hungarian and had valid health insurance. The gender ratio was close to equal, 83 females and 88 males were examined. The mean birth weight of infants was between 260 and 2490 g (M = 1735.56, SD = 579.74). Gestational age of our sample was also low (M = 32.53, SD = 3.27). Main characteristics of our entire sample are shown in Table 1. The same statistics calculated for specific subsamples (for groups with respect to planning and mode of conception) shall be shown and discussed later in the following sections of Results (see Tables 3 and 4).

**Table 1:** Perinatal and socio-demographic characteristics of the overall sample

***Perinatal data***	
Gestational age (weeks), n (%)	
very early (< 28 weeks)	20/171 (11.69)
early (28–31 weeks)	29/171 (16.95)
moderate (32–33 weeks)	42/171 (24.56)
late (34–36 weeks)	80/171 (46.78)
Birth weight (g), n (%)	
Not LBW (> 2500 g)	0/171 (0)
LBW (1500–2499 g)	108/171 (63.15)
VLBW (1000–1499 g)	38/171 (22.22)
ELBW (< 1000 g)	25/171 (14.61)
Mode of delivery, n (%)	
Sectio caesarea	118/171 (69.00)
Per vias naturales	51/171 (29.82)
Apgar scores, M ± SD	
1 min	7.67 ± 1.62
5 min	8.80 ± 1.09
10 min	9.27 ± 0.73
Intensive care, n (%)	
NICU-admission	143/171 (83.62)
Sub-intensive care	19/171 (11.11)
Chronic neonatal morbidities, n (%)	
BPD (bronchopulmonary dysplasia)	12/171 (7.01)
ROP (retinopathy of prematurity)	6/171 (3.50)
IVH (intraventricular haemorrhage)	5/171 (2.92)
NEC (necrotizing enterocolitis)	1/171 (0.58)
PDA (patent ductus arteriosus)	12/171 (7.01)
ASD (atrial septal defect)	2/171 (1.16)
VSD (ventricular septal defect)	2/171 (1.16)
***Socio-demographic data***	
Maternal age at childbirth (years), M ± SD	30.79 ± 6.11
Maternal education, n (%)	
Primary	24/171 (14.03)
Secondary technical education	25/171 (14.61)
Secondary vocational education	26/171 (15.20)
Secondary grammar education	19/171 (11.11)
College	38/171 (22.22)
University	33/171 (19.29)
Marital status, n (%)	
Single	9/171 (5.26)
Married	105/171 (61.40)
Cohabiting	39/171 (22.80)
In a relationship	11/171 (6.43)
Social status, n (%)	
Above-average	68/171 (39.76)
Average	76/171 (44.44)
Below-average	17/171 (9.94)

### Neurodevelopmental outcomes on Bayley-III Screening Test subscales

One of the great benefits of the Bayley-III Screening Test is its ability to predict the risk of developmental delays. It can distinguish high, moderate and low risk for developmental delays compared to levels expected based on their age. In our sample, the proportion of 'high risk for developmental delay' was low, ranging from 0% (cognitive subscale) to 2.92% (expressive language and fine motor subscales). The proportion of ‘moderate risk for developmental delay' was slightly higher, ranging from 11.11% (cognitive subscale) to 27.48% (expressive language subscale) (Table 2).

**Table 2:** Developmental performance on Bayley-III Screening Test subscales (*n* = 171)

*Mean subscale score*		*Risk for Developmental delay*	
	Mean ± SD	'High risk for developmental delay', n (%)	'Moderate risk for developmental delay', n (%)
Cognitive Subscale	16.85 ± 1.45	0/171 (0.00)	19/171 (11.11)
Receptive Language Subscale	11.46 ± 1.62	4/171 (2.33)	35/171 (20.46)
Expressive Language Subscale	10.84 ± 1.87	5/171 (2.92)	47/171 (27.48)
Fine Motor Subscale	12.40 ± 1.55	5/171 (2.92)	34/171 (19.88)
Gross Motor Subscale	15.38 ± 1.80	4/171 (2.33)	30/171 (17.54)

### MAR and neurodevelopmental outcomes, perinatal and socio-demographic characteristics

In our sample 47 (27.48%) children were conceived through medically assisted reproduction with 124 (72.51%) infants conceived naturally, without any medical assistance. In 15 cases we did not get any information about the special type of medically assisted reproduction, only ovulation induction (*n* = 2), artificial insemination (*n* = 6) and IVF (*n* = 24) occurred, so we formed an integrated MAR group. At the level of descriptive statistics (mean ± std. dev.), MAR group (1860.85 ± 407.78 g; 32.87 ± 1.97 weeks) had higher mean values than naturally conceived peers (1684.27 ± 621.16 g; 32.34 ± 3.61 weeks) at the birthweight and gestational age. Examining the Bayley-III Screening Test scores, in the group of infants conceived through MAR and naturally, we found that the neurodevelopment of infants conceived by MAR was above average in all developmental areas compared to naturally conceived peers (Table 3). Significant differences (*p* < 0.05) were found at receptive language and gross motor subscales on both t-tests and Mann–Whitney tests.

**Table 3:** Neurodevelopmental outcomes, perinatal and socio-demographic characteristics of infants conceived by MAR and naturally (means, *p*-values)

	*Mean subscale scores*		*p-values (test statistics)*	
	MAR (*n* = 47)	Natural conception (*n* = 124)	Mann–Whitney test *p*-value (U)	t-test *p*-value (t)
*Neurodevelopmental outcomes*				
Cognitive Subscale	17.10	16.76	0.065 (2393.0)	0.172 (1.371)
Receptive Language Subscale	12.00	11.26	0.004 (2096.5)*	0.003 (3.076)*
Expressive Language Subscale	11.21	10.70	0.101 (2449.0)	0.111 (1.603)
Fine Motor Subscale	12.55	12.34	0.320 (2632.5)	0.441 (0.772)
Gross Motor Subscale	16.02	15.14	0.004 (2114.5)*	0.004 (2.893)*
	*Perinatal and socio-demographic characteristics*		*p-values of t-tests or χ^2 tests*	
*Perinatal data*				
Gestational age (weeks), n (%)			0.135	
very early (< 28 weeks)	2 (4.25)	18 (14.51)		
early (28–31 weeks)	8 (17.02)	20 (16.12)		
moderate (32–33 weeks)	16 (34.04)	26 (20.96)		
late (34–36 weeks)	21 (44.68)	60 (48.38)		
Birth weight (g), n (%)			0.017*	
LBW (1500–2499 g)	34 (72.34)	74 (59.67)		
VLBW (1000–1499 g)	12 (25.53)	26 (20.96)		
ELBW (< 1000 g)	1 (2.12)	24 (19.35)		
Mode of delivery, n (%)			0.234	
Sectio caesarea	36 (76.59)	82 (66.12)		
Per vias naturales	11 (23.40)	40 (32.25)		
Apgar scores, M ± SD				
1 min	8.11 ± 1.34	7.51 ± 1.68	0.037*	
5 min	9.16 ± 0.81	8.67 ± 1.16	0.03*	
10 min	9.5 ± 0.58	9.19 ± 0.76	0.069	
Intensive care			0.516	
NICU-admission, n (%)	41 (87.23)	102 (82.25)		
Sub-intensive care, n (%	5 (10.63)	14 (11.29)		
Chronic neonatal morbidities, n (%)				
BPD	0	12 (9.67)	–	
ROP	0	6 (4.83)	–	
IVH	2 (4.25)	3 (2.41)	–	
NEC	1 (2.12)	0	–	
PDA	1 (2.12)	11 (8.87)	–	
ASD	0	2 (1.61)	–	
VSD	0	2 (1.61)	–	
*Socio-demographic data*				
Maternal age at childbirth (years), M ± SD	32.78 ± 4.61	30.04 ± 6.44	0.003*	
Maternal education, n (%)			0.035*	
Primary	2 (4.25)	22 (17.74)		
Secondary technical education	4 (8.51)	21 (16.93)		
Secondary vocational education	6 (12.76)	20 (16.12)		
Secondary grammar education	5 (10.63)	14 (11.29)		
College	15 (31.91)	23 (18.54)		
University	14 (29.78)	19 (15.32)		
Marital status, n (%)			0.030*	
Single	0	9 (7.25)		
Married	34 (72.34)	71 (57.25)		
Cohabiting	12 (25.53)	27 (21.77)		
In a relationship	0	11 (8.87)		
Social status, n (%)			0.043*	
Above-average	26 (55.31)	42 (33.87)		
Average	15 (31.91)	61 (49.19)		
Below-average	6 (12.76)	11 (8.87)		

^*^ *p* < 0.05, in case of t-test either the independent sample one or the Welch statistic. Note that for some variables the joint frequencies are very low or equal to zero in several (too many) cells, hence the test and its *p*-value would not be meaningful in case of such an asymptotic test. In these cases (denoted by –) we omit to show the *p*-values

Furthermore, we have examined the perinatal and socio-demographic differences between the MAR and the naturally conceived groups, using similar statistics and setup as in Table 1 for the entire sample (see Table 3). We identified significant (*p* < 0.05) differences in birthweight, 1 min and 5 min Apgar scores, maternal age, maternal education, marital and social status. Infants conceived by MAR had higher Apgar-scores and their mothers were older in average. Having a look at the percentage relative frequency distribution, we found higher proportions in case of LBW and VLBW infants in the MAR group, whereas mothers in the MAR group had a lower incidence of low and secondary education and a higher incidence of college and university education. Furthermore, proportions of marriage, cohabiting as well as the above-average social status are higher than in the group of naturally conceived peers (Table 3).

### Pregnancy planning and neurodevelopment outcomes, perinatal and socio-demographic characteristics

According to the parental interviews, 87.13% of the infants (*n* = 149) were intended pregnancies. We identified unplanned pregnancies in 17 cases (9.94%) and we did not get any information about pregnancy planning in 5 cases (2.92%). Examining the mean birth weight and gestational age of planned (1740.53 ± 579.08 g; 32.41 ± 3.28 weeks) and unplanned (1562.35 ± 527.63 g; 32.41 ± 3.14 weeks) infants we identified higher birth weight in the planned group. Examining the subscales of Bayley Screening Test, planned infants showed higher scores for all variables. The Mann–Whitney test showed significant differences (*p* < 0.05) between planned and unplanned pregnancies on cognitive, receptive and expressive language subscales. T-tests showed similar results, significant differences were identified on the cognitive, receptive language and gross motor subscale levels (Table 4).

**Table 4:** Neurodevelopmental outcomes, perinatal and socio-demographic characteristics of planned and unplanned infants (means, *p*-values)

	*Mean subscale scores*		*p-values (test statistics)*	
	Planned (*n* = 149)	Unplanned (*n* = 17)	Mann–Whitney test *p*-value (U)	t-test *p*-value (t)
*Neurodevelopmental outcomes*				
Cognitive Subscale	16.95	16.17	0.027 (861.5)*	0.036 (2.109)*
Receptive Language Subscale	11.63	10.17	0.001 (629.5)*	0.000 (3.623)*
Expressive Language Subscale	10.93	10.05	0.015 (816.5)*	0.069 (1.830)
Fine Motor Subscale	12.46	12.05	0.269 (1063.5)	0.312 (1.014)
Gross Motor Subscale	15.46	14.52	0.089 (956.5)	0.044 (2.029)*
	*Perinatal and socio-demographic characteristics*		*p-values of t-tests or χ^2 tests*	
*Perinatal data*				
Gestational age (weeks), n (%)			0.892	
very early (< 28 weeks)	18 (12.08)	2 (11.76)		
early (28–31 weeks)	24 (16.10)	4 (23.52)		
moderate (32–33 weeks)	37 (24.83)	4 (23.52)		
late (34–36 weeks)	70 (46.97)	7 (41.17)		
Birth weight (g), n (%)			0.324	
LBW (1500–2499 g)	96 (64.42)	8 (47.05)		
VLBW (1000–1499 g)	31 (20.80)	6 (35.29)		
ELBW (< 1000 g)	22 (14.76)	3 (17.64)		
Mode of delivery, n (%)			0.497	
Sectio caesarea	102 (68.45)	13 (76.47)		
Per vias naturales	47 (31.54)	4 (23.52)		
Apgar scores, M ± SD				
1 min	7.68 ± 1.55	7.26 ± 2.18	0.340	
5 min	8.78 ± 1.11	8.73 ± 0.96	0.869	
10 min	9.25 ± 0.74	9.2 ± 0.63	0.839	
Intensive care			–	
NICU-admission, n (%)	125 (83.89)	15 (88.23)		
Sub-intensive care, n (%	16 (10.73)	1 (5.88)		
Chronic neonatal morbidities, n (%)				
BPD	12 (8.05)	0	–	
ROP	5 (3.35)	1 (5.88)	–	
IVH	5 (3.35)	0	–	
NEC	1 (0.67)	0	–	
PDA	11 (7.38)	1 (5.88)	–	
ASD	0	2 (11.76)	–	
VSD	1 (0.67)	1 (5.88)	–	
*Socio-demographic data*				
Maternal age at childbirth (years), M ± SD	31.34 ± 5.71	26.94 ± 7.80	0.037*	
Maternal education, n (%)			0.159	
Primary	17 (11.40)	5 (29.41)		
Secondary technical education	21 (14.09)	4 (23.52)		
Secondary vocational education	23 (15.43)	3 (17.64)		
Secondary grammar education	17 (11.40)	2 (11.76)		
College	37 (24.83)	1 (5.88)		
University	32 (21.47)	1 (5.88)		
Marital status, n (%)			0.000*	
Single	3 (2.01)	4 (23.52)		
Married	101 (67.78)	4 (23.52)		
Cohabiting	33 (22.14)	6 (35.29)		
In a relationship	9 (6.04)	2 (11.76)		
Social status, n (%)			–	
Above-average	67 (44.96)	1 (5.88)		
Average	63 (42.28)	11 (64.70)		
Below-average	16 (10.73)	1 (5.88)		

^*^ *p* < 0.05, in case of t-test either the independent sample one or the Welch statistic. Note that for some variables the joint frequencies are very low or equal to zero in several (too many) cells, hence the test and its *p*-value would not be meaningful in case of such an asymptotic test. In these cases (denoted by –) we omit to show the *p*-values

As in the previous section, we have examined the perinatal and socio-demographic differences again, this time between the planned and unplanned groups, of course (see Table 4). We identified significant (*p* < 0.05) differences in maternal age and marital status. Mothers in the unplanned group were younger in average; analysing the percentage relative frequencies one can see that single marital status was higher, while marriage was lower than in the planned group. Note further that the percentage relative frequency distributions seem to be different in the planned and unplanned groups e.g. in case of the variables birth weight and maternal education, that is the proportions of VLBW and ELBW infants, the low and secondary education are all higher in the unplanned group, but presumably there is no significant difference due to the small sample size (*n* = 17, see Table 4).

### Other explanatory factors of neurodevelopmental outcomes

ANOVA was used to determine whether pregnancy planning and mode of conception were the most decisive factors or other variables influenced cognitive, language and motor development. We pre-screened those variables that we found relevant and available in this regard, such as gestational age, birth weight, participation in developmental interventions, marital status, maternal education, housing, social status and income. Subsequently, the relationship between the pre-screened variables and neurodevelopment was investigated with suitable tests: in the case of nominal variables both with t-tests (as before) and One-Way ANOVA and with their non-parametric counterparts, Mann–Whitney and Kruskal–Wallis tests. For some variables, where there were appropriate scales, the relationship was tested with the existence of a significant correlation. In case of marital status, maternal education, housing, social status, and income variables no significant relationship was identified using the Bayley-III Screening Test subscales, so these variables were excluded from further analyses. Five independent variables (factors) (gestational age, birth weight, participation in developmental interventions, pregnancy planning and mode of conception) were considered, which we hypothesized they may influence – at least or have relationship with – the neurodevelopmental variables. These five variables were our explanatory factors in ANOVA, such that interactions were also included in the model. It would be interesting to extend our analysis with further determinants, such as BPD, IVH, NEC, or ROP. However, one can see the very low frequency values for the positive cases of these neonatal morbidities in the sample (see Table 1); hence, we could not consider such an extended analysis.

According to the results of ANOVA, no significant relationship could be identified on the cognitive or fine motor subscales in case of the five factors. Concerning the receptive language subscale, the pregnancy planning variable had a significant role (F = 9.513, *p* = 0.002). For the expressive language subscale, MAR showed a weak, but significant relationship (F = 2.902; *p* = 0.090) as it can be detected only with a first-order error (*p* < 0.01). Interaction of gestational age with the MAR variable had an explanatory power on the expressive language subscale (F = 3.491; *p* = 0.017). Concerning the gross motor subscale, birth weight (F = 9.352; *p* = 0.000) and pregnancy planning (F = 6.132; *p* = 0.014) variables had an independent and joint impact (F = 2.629; *p* = 0.075). The mean neurodevelopmental performance of each group is shown in Tables 5 and 6.

**Table 5:** Descriptive statistics by gestational age, pregnancy planning and development on Language Subscales: (n), M ± SD

*Receptive Language Subscale*			*Expressive Language Subscale*		
Gestational age	Pregnancy planning and participation in developmental interventions (n)	Mean ± SD	Gestational age	Mode of conception (n)	Mean ± SD
Late	Planned				
	developed (21)	12.09 ± 1.33	Late	MAR (21)	11.71 ± 1.58
	not developed (49)	11.02 ± 1.45		natural (60)	10.61 ± 2.04
	Unplanned				
	developed (2)	10.00 ± 1.41	Moderate	MAR (16)	10.12 ± 1.58
	not developed (5)	10.20 ± 1.64		natural (26)	11.19 ± 1.38
Moderate	Planned				
	developed (17)	11.41 ± 1.46	Early	MAR (8)	11.87 ± 1.12
	not developed (20)	11.70 ± 1.26		natural (20)	10.85 ± 2.10
	Unplanned				
	not developed (4)	11.00 ± 0.81	Very early	MAR (2)	12.00 ± 0.00
Early	Planned			natural (18)	10.11 ± 1.99
	developed (13)	12.38 ± 2.06			
	not developed (11)	11.81 ± 1.47			
	Unplanned				
	developed (1)	11.00			
	not developed (3)	8.33 ± 1.15			
Very early	Planned				
	developed (13)	11.92 ± 2.06			
	not developed (5)	13.00 ± 0.70			
	Unplanned				
	developed (1)	12.00			
	not developed (1)	10.00

**Table 6:** Descriptive statistics by birth weight and pregnancy planning on Gross Motor Subscale: (n) M ± SD

*Gross Motor Subscale*		
Birth Weight	Pregnancy planning (n)	Mean ± SD
ELBW	planned (22)	13.90 ± 1.23
	unplanned (3)	12.33 ± 1.52
VLBW	planned (31)	16.00 ± 1.59
	unplanned (6)	14.00 ± 2.36
LBW	planned (96)	15.65 ± 1.72
	unplanned (8)	15.75 ± 1.66

## Discussion

### Neurodevelopmental outcomes (cognitive, receptive and expressive language, fine and gross motor)

The Bayley-III Screening Test provides comprehensive information of early childhood cognitive, language and motor development. It is also able to screen children at risk of delayed development, and distinguish high, moderate or low risk for developmental delays. However, the Screening Test does not replace the full BSID-III assessment, based on its results; children cannot be considered to be late in development but only at risk for development delay [38]. In our study, only preterm infants at 12 months of corrected age were examined, we did not have a full-term control group. According to the Bayley-III Screening Test results, receptive, expressive language and fine motor subscales showed the lowest scores and were the most outstanding in terms of risk for developmental delay. This result is consistent with literature findings, regarding preterm infants having significantly lower neurodevelopmental (cognitive, language and motor) skills [42, 43]. Presumably, motor problems negatively affect communication skills, such as face-to-face interaction, joint attention and early linguistic acquisition [13, 42]. Severe or mild mental and neurodevelopmental delay [44], internalising and externalising problems, somatic symptoms [45], behavioural and attention problems, lower academic achievement (mathematics, reading and spelling skills) [46] can be identified in greater proportions of preterm infants in later life. Screening preterm babies at the earliest age, identifying factors that positively or negatively affect their development and initiating early interventions are major aspects of public health.

### Mode of conception

Comparing research on the development of infants conceived by assisted reproduction with other research is difficult, not only because of the age of the children involved but also due to the variation of the methodological tools used [47, 48]. According to the systematic review of Bay et al. [49], numerous studies have shown no deficits in neurodevelopmental abilities of children conceived through assisted reproduction compared to naturally conceived counterparts [50–53]. However, there is no complete consensus in the literature. Few studies have identified lower neurodevelopmental performance at 12 and 24 months of age [54, 55], however these findings are discussed. Prospective cohort study of Minh Tuan Vo et al. [56] showed that IVF children at 5 through 30 months have slower language and motor development. On the contrary, Schendelaar et al. [57] identified better fluency of movements at 24 months of age, while Carson et al. [47] described higher verbal skills of children born after assisted reproduction at 3 and 5 years of age. Barbuscia et al. [58] also identified higher verbal abilities at ART children at the age of 3 and 5 years. However, their results suggest that the positive ‘ART effect’ is inseparable from parental characteristics, like higher socioeconomic status, better education and older age. Socioeconomic status usually includes the education and occupation of parents, the family structure, the income, and the ethnicity of the family [59]. From these variables, we analysed the maternal age and education, the marital and social status. Our findings are consistent with the results of Barbuscia et al. [58]; we identified higher social status, educational level and maternal age in the mothers of MAR infants. Concerning cognitive skills, many researchers found no difference in development [48, 60], while others have identified lower developmental outcomes [61, 62]. Balayla et al. [63] compared the main Bayley-III subscales of ART and naturally conceived infants at 2 years, but the groups had similar cognitive, language and motor outcomes.

Based on our study results, infants who were conceived by MAR performed not only similarly but also much better (significantly on the receptive language and gross motor subscales) than naturally conceived children. According to the results of the ANOVA technique, the mode of conception had an independent role on the expressive language subscale, and we also found explanatory power in interaction with gestational age. We can assume that these children may have experienced greater parental attention and care from the moment of conception; factors that may have contributed to higher cognitive, language and motor development at 12 months of corrected age. Based on the literature findings parents may perceive their infants more fragile due to the health outcomes (e.g. increased risk of preterm birth and LBW), the long conception procedure, so they pay more attention and care of them, which can be reflected in higher neurodevelopmental outcomes in the early years of life. The financial and emotional efforts to have a baby conceived by MAR can also contribute to the higher care in parenting [47, 58].

### Pregnancy planning

Review of studies about pregnancy planning shows that there is relatively scarce international research where long-term developmental pathways are followed. This line of our research is considered completely groundbreaking in Hungary. Many factors can adversely affect the development of a child born from an unplanned/unintended pregnancy [64]. Carson et al. [47] found that children born of unintended pregnancies have lower verbal skills at 3 or 5 years of age than their planned or artificially conceived counterparts, however this difference was explained by socioeconomic inequalities. In kindergarten, these children show lower social-emotional development [32]. Baydar [65] identified lower receptive vocabulary of children younger than two did, while de La Rochebrochard and Joshi [66] identified poorer developmental scores and cognitive delay in children born unplanned pregnancies at 3 years.

Our results show significantly lower language skills already at 12 months of corrected age, as well as significantly lower cognitive skills. With ANOVA, we examined whether differences in neurodevelopmental performance can be explained by pregnancy planning. In the cognitive subscale, pregnancy planning was not considered significant. In case of the receptive language subscale, pregnancy planning had an independent impact on performance, while in the gross motor subscale birth weight and pregnancy planning had both an independent and interactive role on the subscale. Although we can identify higher mean scores of planned infants, it cannot be concluded that pregnancy planning is solely responsible for all differences. However, it must be emphasized that pregnancy planning alone had a significant role on receptive language and gross motor subscales.

### Limitations

Potential limitations of our study are the small sample size in certain groups; however, our sample covers all available cases in the Eastern region of Hungary. Other limitations are the absence of a full-term control group, and the fact the emotions related to conception and time from pregnancy planning to conception were not measured. To overcome these limitations we examine our sample’s development with the full BSID-III assessment at the 24 months of corrected age, ask more detailed information about the circumstances of pregnancy planning and gather an adjusted full-term control group.

With our statistical analysis, we cannot unequivocally conclude that MAR and pregnancy planning had the main role on the neurodevelopment of preterm infants at 12 months of corrected age, we can only determine a positive association between variables by the statistical tests, and not necessarily a causal relationship. Though we note again that some (socio-demographic) variables explaining partially the parental characteristic was included in the analysis but have been found not to have any significant explanatory power when we took into account the role of other factors (see ANOVA results) jointly.

## Conclusions

In terms of neurodevelopmental and intellectual development, preterm children can be regarded as a vulnerable population [44, 45]. Unplanned pregnancies and medically assisted reproduction are often associated with higher rates of preterm births and low birth weight [21, 22]. Cognitive, language and motor screening of children at risk for developmental delay (e.g. children conceived by MAR, preterm and unplanned infants) is essential. Bayley-III Screening Test is an excellent tool for monitoring developmental skills in the early years of life. In addition to providing a complete picture of the child's early cognitive, receptive and expressive language, as well as fine and gross motor skills, it helps to identify problematic areas, making early interventions possible. The potential short- and long-term unfavourable outcomes can be reduced. The significance of our study is increased by the fact it is one of the first pieces of research into preterm infants conducted in Hungary using Bayley-III Screening Test. With our examinations, we wish to promote the importance of early psychological screening. Early detection and treatment of developmental problems can have great benefits not only for the individual but also for society.

## Data Availability

Not applicable.

## Abbreviations

ART: Assisted reproductive technology
ASD: Atrial septal defect
BPD: Bronchopulmonary dysplasia
BSID-II: Bayley Scales of Infant Development – Second Edition
BSID-III/Bayley-III: Bayley Scales of Infant and Toddler Development – Third Edition
CHD: Congenital heart defects
CLD: Chronic lung disease
ELBW: Extremely low birth weight
ICMART: International Committee Monitoring Assisted Reproductive Technologies
ICSI: Intracytoplasmic sperm injection
IUI: Intrauterine insemination
IVF: In vitro fertilization
IVH: Intraventricular haemorrhage
LBW: Low birth weight
MAR: Medically assisted reproduction
NEC: Necrotizing enterocolitis
NICU: Neonatal Intensive Care Unit
PDA: Patent ductus arteriosus
ROP: Retinopathy of prematurity
VLBW: Very low birth weight
VSD: Ventricular septal defect
